# Mrc1 protects uncapped budding yeast telomeres from exonuclease *EXO1*

**DOI:** 10.1016/j.dnarep.2007.05.010

**Published:** 2007-11-01

**Authors:** Avgi Tsolou, David Lydall

**Affiliations:** Centre for Integrated Systems Biology of Ageing and Nutrition, Institute for Ageing and Health, Henry Wellcome Laboratory for Biogerontology Research, Newcastle University, Newcastle upon Tyne NE4 6BE, UK

**Keywords:** Mrc1, Telomere, ssDNA, *CDC13*, *YKU70*, *EXO1*

## Abstract

Mrc1 (Mediator of Replication Checkpoint 1) is a component of the DNA replication fork machinery and is necessary for checkpoint activation after replication stress. In this study, we addressed the role of Mrc1 at uncapped telomeres. Our experiments show that Mrc1 contributes to the vitality of both *cdc13-1* and *yku70Δ* telomere capping mutants. Cells with telomere capping defects containing *MRC1* or *mrc1*^*AQ*^, a checkpoint defective allele, exhibit similar growth, suggesting growth defects of *cdc13-1 mrc1Δ* are not due to checkpoint defects. This is in accordance with Mrc1-independent Rad53 activation after telomere uncapping. Poor growth of *cdc13-1* mutants in the absence of Mrc1 is a result of enhanced single stranded DNA accumulation at uncapped telomeres. Consistent with this, deletion of *EXO1*, encoding a nuclease that contributes to single stranded DNA accumulation after telomere uncapping, improves growth of *cdc13-1 mrc1Δ* strains and decreases ssDNA production. Our observations show that Mrc1, a core component of the replication fork, plays an important role in telomere capping, protecting from nucleases and checkpoint pathways.

## 1 Introduction

Telomeres are specialized DNA–protein complexes at the end of eukaryotic chromosomes. Proper telomere structure is essential for chromosome integrity and genome stability because telomeres protect natural chromosome ends from degradation and end-to-end fusion and because they ensure complete genome replication. Telomeres differ from Double Strand Breaks (DSBs) in that they normally fail to activate DNA repair or DNA damage checkpoint pathways. If that was the case, then they would undergo recombination and chromosomal fusions and dicentric chromosomes would be generated [1–4].

Many proteins associate with telomeric DNA. These proteins ensure that telomeres behave differently from DSB ends and help maintain chromosomal stability. Some telomeric proteins bind specifically to dsDNA and others show higher affinity to ssDNA. In budding yeast, there are numerous proteins with affinity for telomeric dsDNA, such as Rap1, Sir2, Sir3, Sir4, Rif1 and Yku70/Yku80 [5]. The budding yeast telomeric ssDNA ends are thought to be protected by three essential proteins, Cdc13, Stn1 and Ten1 [6–11].

If telomeres become uncapped, they activate a DNA damage response pathway leading to cell cycle arrest [12–14]. Moreover, recently it has been suggested that telomeres trigger a transient DNA damage response in each S phase in order to complete DNA replication and cap chromosome ends [15]. DNA damage response pathways are complex networks which include – among other components – mediators. Mediators facilitate the transmission of the DNA damage signal from sensors to downstream effectors; activation of the latter affecting cell cycle progression [16]. Mrc1 (Mediator of Replication Checkpoint 1) appears to take the mediator role in Rad53 activation under replication stress [17]. However, parallel pathways exist because in *mrc1Δ* mutants Rad53 activation occurs through Rad9 (another mediator protein), presumably because the accumulation of “DNA damage” rather than “replication defects” in *mrc1Δ* mutants leads to activation of Rad9 and thereby activation of Rad53 [17].

Mrc1 also appears to be directly involved in DNA replication and, because of this, *mrc1Δ* cells display a slow S phase [18]. Mrc1 is an active component of the replication machinery, loaded onto DNA shortly after replication initiates, and moving with other components of replication forks [19–21]. In the presence of hydroxyurea, a type of replication stress, Mrc1 appears to form a stable replication-pausing complex preventing the uncoupling of the replication machinery from DNA synthesis [20–22]. According to this model, Mrc1 mediates activation of Rad53 under conditions of replication stress so that subsequent DNA repair events occur and cell replication resumes normal function [22]. However, recent experiments suggest that the role of Mrc1 at stalled replication forks is more than activating Rad53, since *mrc1*^*AQ*^ cells, defective in Rad53 activation, are not defective in replication fork initiation or progression [18,20,21]. *mrc1*^*AQ*^ is a mutant allele in which SQ/TQ residues have been substituted with AQ, resulting in its inability to mediate phosphorylation and activation of Rad53 [18].

Although Mrc1 is involved in the DNA replication checkpoint, it has been shown that it is not required for the DNA damage checkpoint, since *cdc13-1 mrc1Δ* double mutants arrest in G2 at non-permissive temperatures [17]. It has been reported that activation of Rad53 in response to telomere shortening still occurs in the absence of Rad9 and that Mrc1 is responsible for this activation in telomerase-deficient cells, in which telomeres continually shorten until they activate a checkpoint [23]. Surprisingly, though, *tlc1*Δ *mrc1*Δ double mutants arrest cell division, suggesting that Mrc1 is not required for cell cycle arrest in telomerase negative cells. In contrast, after *cdc13-1* induced telomere uncapping, Rad53 activation is entirely Rad9-dependent and Mrc1-independent [23].

Here we investigated the role of Mrc1 at uncapped telomeres, using the temperature sensitive *cdc13-1* and *yku70Δ* mutations to uncap telomeres. Our experiments indicate that Mrc1 protects telomeres from the DNA damage response and that the role of Mrc1 in DNA replication forks, rather than in checkpoint activation, is important for protection of telomeres.

## 2 Materials and methods

### 2.1 Yeast strains and plasmids

All strains in the W303 background are *RAD5* and they contain an *ade2-1* mutation (Supplemental Table 1); therefore yeast extract/peptone/dextrose (YEPD) was supplemented with adenine at 50 mg/l. Strains 3393–3402 are in the S288C background (Supplemental Table 1) and they were generated by mating a single gene deletion mutant array [24] with a *cdc13-1* query strain [25]. To construct strains, standard genetic procedures of transformation and tetrad analysis were used [26]. p*MRC1* and p*mrc1*^*AQ*^, also carrying the *URA3* gene were a gift from Steven Elledge [17,18].

*MRC1* was disrupted in two different ways. Firstly, the *MRC1* ORF was substituted with *KanMX6*, with a PCR based method [27]. Primers 5′-tcgttattcgcttttgaacttatcaccaaatattttagtgCGGATCCCCGGGTTAATTAA-3′ (#878) and 5′-ctggagttcaatcaacttcttcggaaaagataaaaaaccaGAATTCGAGCTCGTTTAAAC-3′ (#881), which contain homology to upstream and downstream sequences of *MRC1* (bases in lowercase), were used to amplify a 1559 bp *KanMX*6 sequence (pFA6a-kanMX6; [27]). The PCR product was transformed into yeast and candidate colonies were selected for G418 resistance. Integration of the *KanMX*6 marker into the *MRC1* locus was confirmed by PCR, using two sets of primers: (i) forward 5′-CCAAGAACAGACAAACAACTAAGGA-3′ (#876) with reverse primer 5′-TCAGCATCCATGTTGGAATT-3′ (#81) and (ii) forward 5′-CCATCCTATGGAACTGCCTC-3′(#82) with reverse 5′-CCTAGACTCGGGTGCCATCT-3′ (#880). Disruption of *MRC1* was also confirmed by Southern blot (data not shown). Alternatively, *MRC1* was substituted with *URA3* using a restriction enzyme digest approach. First, p*MRC1* was digested with XhoI and a 5008 bp fragment containing the full *MRC1* gene was cloned into XhoI digested pIC19H vector (2.7 kb) to create pAT1065. A correct clone was identified by restriction digests. pAT1065 was digested with SpeI to remove a 2.31 kb DNA fragment containing the bulk of *MRC1*, which was replaced with a 1.3 kb BamHI *URA3* gene fragment from pDL349 (pBSB + KS containing a BamHI fragment containing the *URA3* gene) by blunt cloning following treatment with DNA polymerase I Large (Klenow) fragment (New England Biolabs). Positive clones were selected by restriction enzyme digests to identify the disruption of the bulk of *MRC1* with *URA3* (pAT1066). Disruption of *MRC1* was also confirmed by Southern blot (data not shown). To disrupt *MRC1*, pAT1066 was digested with XhoI prior to transformation of yeast.

### 2.2 Spot tests

Single colonies were inoculated into 2 ml YEPDextrose (YEPD) and incubated overnight, with aeration, at 23 °C. The following day, 200 μl of each culture was inoculated into 2 ml of fresh YEPD and returned to 23 °C. Cells were grown for three more hours, and cell numbers were determined in a haemocytometer. The cells were then centrifuged (13,000 rpm for 10 s in a microcentrifuge), washed twice with sterile water and resuspended in water to a final concentration of 1.5 × 10^7^ cells/ml. A five-fold dilution series of each of the cultures was prepared using sterile water in a 96 well plate and 3–5 μl spotted onto plates using a 48-prong replica plating device. Plates were incubated at various temperatures for 2–3 days before being photographed. For spot tests with strains containing p*MRC1*, p*mrc1*^*AQ*^ or pRS416 the steps were as described above, but strains were grown on selective medium (-URA). All strains shown as if on a single agar plate were grown on the same plate, although in some cases their positions were moved using Adobe Photoshop and Adobe Illustrator CS. Unless otherwise stated, at least two different strains of the same genotype were spot tested and representative examples are shown.

### 2.3 Yeast transformation

High efficiency transformations needed for gene disruptions were performed according to Gietz et al. [28]. For plasmid transformations a more rapid method was used [29].

### 2.4 Western blots

Protein extracts were prepared by glass bead breakage in TCA, essentially as previously described [30,31]. Bio-Rad gels (7.5% Tris–HCl), Schleicher and Schuell Protan Nitrocellulose membranes and the Pierce Supersignal West Pico Chemiluminescent Substrate detection kit were used in a standard Western blot procedure. Rabbit anti-Rad53 polyclonal antibody (AbDL50, 1:1000 dilution, a gift from Dan Durocher [32]) was a primary antibody used with an anti-rabbit-HRP (AbDL7, 1:10,000 dilution, Dako P0448) as a secondary antibody used. Mouse anti-tubulin (TAT-1, AbDL42, 1:2000 dilution, a gift from Keith Gull [33] and anti-mouse-HRP (AbDL6, 1:10,000 dilution, Dako P0447) were used for tubulin loading controls.

### 2.5 Telomere length measurement by Southern blot

Strains were grown to saturation in liquid YEPD at 23 °C. DNA from each strain was subjected to XhoI cut. The digested DNA was loaded on a 0.8% agarose gel, run at low voltage overnight, transferred to a Magna nylon membrane and UV cross-linked. The membrane was then hybridised with a Y′-TG probe [34]. A non-radioactive detection kit was used for the detection of the hybridisation (Amersham, Arlington Heights, IL).

### 2.6 Synchronous cultures

*cdc13-1 cdc15-2 bar1*Δ strains with additional mutations (see Supplemental Table 1) were grown in YEPD at 23 °C overnight. In the morning, cells were adjusted to a concentration of 8 × 10^6^ buds/ml in 250 ml. Cultures were grown for three more hours, then arrested with 20 nM α-factor for a further 2.5 h. The cultures were then released from G1 arrest by centrifugation and washed twice in YEPD and placed at 36 °C, 40 min after the culture was first centrifuged. Cell cycle position was monitored as previously described [13]. DNA was prepared and the fraction of single stranded DNA (ssDNA) was measured as previously described [35].

### 2.7 Asynchronous cultures

*cdc13-1* strains with additional mutations indicated were grown in YEPD at 23 °C overnight. In the morning, cells were adjusted to a concentration of 1 × 10^7^ cells/ml and temperature was raised to 27.3 °C. Every 90 min samples were taken for cell cycle position and cell density was re-adjusted to 1 × 10^7^ cells/ml. Cell numbers were determined with a haemocytometer. Samples for Western blots were collected from exponentially growing cultures 2 h after the temperature was raised from 23 °C to 36 °C.

### 2.8 Cell cycle position determination

A 1 ml sample of culture was centrifuged for 8–10 s at high speed, the supernatant was aspirated, and cells were fixed at 70% ethanol overnight. The fixed cells were washed twice in water before being resuspended in 4,6-diamidino-2-phenylindole (DAPI, 0.2 μg/ml). Cell cycle distribution was monitored by DAPI staining of nuclei and fluorescence microscopy. For DAPI staining, 100 cells for each sample were counted and classified as: (1) unbudded, single DAPI-stained body; (2) small budded, single DAPI-stained body, with the bud <50% of the diameter of the mother cell; (3) medial nuclear division, single DAPI-stained body, with bud >50% diameter of mother cell, the *cdc13-1* arrest point; (4) late nuclear division, two buds, and two DAPI-stained bodies, the *cdc15-2* arrest point [36].

## 3 Results

### 3.1 Mrc1 contributes to the vitality of *cdc13-1* and *yku70Δ* mutants

**Fig. 1:**
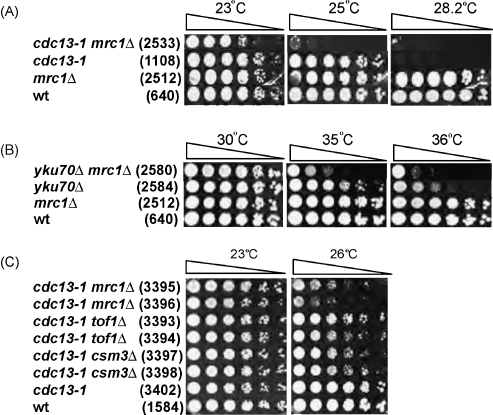
Mrc1 contributes to the vitality of *cdc13-1* and *yku70Δ* mutants. Small aliquots of five-fold dilution series of the strains indicated, and growing at 23 °C, were transferred to plates and incubated at the temperatures shown for 3 days before being photographed. The relevant genotypes are indicated on the left, and strain numbers are shown in parentheses. (A) Growth of W303 *cdc13-1* mutants. (B) Growth of W303 *yku70Δ* mutants. (C) Growth of S288C *cdc13-1* mutants.

Since Mrc1 plays a role in the checkpoint response to stalled replication, we wondered if it also plays a role at uncapped telomeres. The temperature sensitive *cdc13-1* mutation causes a defect in Cdc13, a telomere binding protein, and cells containing this mutation accumulate large amounts of ssDNA at telomeres at non-permissive temperatures [12,37,38]. Interestingly, deletion of checkpoint proteins, like Chk1, Mec1, Mec3, Rad9, Rad17, Rad24 and Rad53 improves growth of *cdc13-1* strains at semi-permissive temperatures [39–41]. This is presumably because checkpoint pathways inhibit cell division by responding to low levels of ssDNA that accumulates at telomeres at semi-permissive temperatures. Deletion of other checkpoint proteins, like the MRX complex, which appears to play a role in telomere capping, worsens the growth of *cdc13-1* strains [42]. Therefore, we wanted to investigate whether Mrc1 plays a role at uncapped telomeres and, if so, whether it behaved like Rad9 or MRX. Fig. 1A shows that deletion of *MRC1* dramatically reduces the growth of *cdc13-1* mutant strains at 25 °C. The effect of Mrc1 is not as profound as that of the MRX complex, as *cdc13-1 mre11Δ* and *cdc13-1 rad50Δ* display more severe growth defects than *cdc13-1 mrc1Δ* even at 23 °C (Supplementary Fig. 1). Thus, Mrc1, like MRX, but unlike the majority of checkpoint proteins, contributes to the vitality of *cdc13-1* strains.

We next wanted to investigate whether analogous growth defects of *cdc13-1 mrc1Δ* strains occur in *yku70Δ* strains. Yku70 is a telomere capping protein which is also involved in dsDNA damage repair and in Non-Homologous End Joining (reviewed in Ref. [43]). Deletion of *YKU70* results in a temperature sensitive phenotype at 37 °C, due to telomere uncapping, which activates a Chk1-dependent cell cycle arrest [36]. Fig. 1B demonstrates that deletion of *MRC1* results in a severe growth defect of *yku70Δ* mutant strains at 35 °C and 36 °C. Thus, Mrc1 contributes to the vitality of *yku70Δ* strains. We conclude that Mrc1 contributes to the vitality of two cell types defective in telomere capping.

Mrc1, Tof1 and Csm3 are three proteins that play similar, although distinct roles in DNA replication [19–21,44]. Therefore, we wished to address whether Tof1 and Csm3, like Mrc1, contributed to the vitality of *cdc13-1* mutants. Spot test analysis showed that although deletion of *TOF1* or *CSM3* also confers some growth defects on *cdc13-1* mutants, deletion of *MRC1* has a stronger phenotype (Fig. 1C). Therefore, we decided to focus on understanding the role of *MRC1* at uncapped telomeres.

### 3.2 Growth defects of *cdc13-1 mrc1Δ* cells are not due to checkpoint defects

In budding yeast, two independent roles have been previously reported for Mrc1. One role implicates Mrc1 as a mediator of checkpoint activation under replication stress and the other role is as part of the replication machinery [17–21]. Therefore, we investigated whether the heightened temperature sensitivity phenotype of *cdc13-1 mrc1Δ* mutant strains is a result of a replication defect, a checkpoint defect or both.

**Fig. 2:**
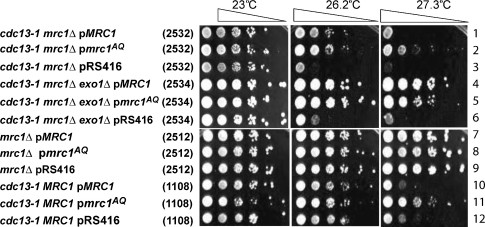
The checkpoint role of Mrc1 is not important for the vitality of *cdc13-1* strains. Small aliquots of five-fold dilution series of the strains indicated, and growing at 23 °C, were transferred to plates and incubated at the temperatures shown for 3 days before being photographed. The relevant genotypes are indicated on the left, and strain numbers are shown in parentheses. Row numbers are designated on the right.

*cdc13-1 mrc1Δ* mutants were complemented with either wild type p*MRC1*, p*mrc1*^*AQ*^ or an empty vector (pRS416) and strains were grown at various temperatures. Fig. 2 shows that at 26.2 °C, complementation of *mrc1Δ cdc13-1* mutant strains, with either p*MRC1* or p*mrc1*^*AQ*^ allele improves growth compared to the empty vector control (compare rows 1–3). Thus, we conclude that the checkpoint role of Mrc1 is not important for the vitality of *cdc13-1* strains.

At higher temperature we noticed an increased growth of *cdc13-1 mrc1Δ* cells carrying the *mrc1*^*AQ*^ allele, compared to p*MRC1*. However, this phenotype was observed even in the presence of the wild type *MRC1* allele, suggesting that this effect is due to some type of dominant effect of *mrc1*^*AQ*^ (Fig. 2, compare rows 2 and 11).

### 3.3 Exo1 inhibits growth of *cdc13-1 mrc1Δ* and *yku70Δmrc1Δ* mutants

**Fig. 3:**
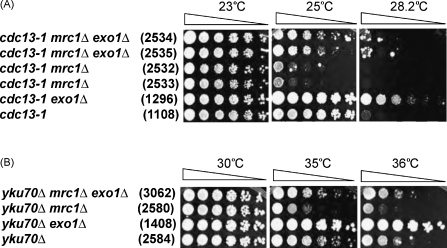
Exo1 contributes to the poor growth of *cdc13-1 mrc1Δ* and *yku70Δ mrc1Δ* strains. Colonies were plated as described in Fig. 1. The relevant genotypes are indicated on the left, and strain numbers are shown in parentheses.

*EXO1* encodes a nuclease known to contribute to ssDNA production at uncapped telomeres of *cdc13-1* and *yku70Δ* strains [36,39]. If Exo1-dependent ssDNA production at uncapped telomeres is responsible for the poor growth of *cdc13-1 mrc1Δ* and *yku70Δ mrc1Δ* mutants, then removing Exo1 should suppress their poor growth. Fig. 3A demonstrates that at 25 °C, *cdc13-1 mrc1Δ exo1Δ* triple mutants exhibit better growth than *cdc13-1 mrc1Δ* strains, showing that Exo1 contributes to the growth defects observed in *cdc13-1 mrc1Δ* strains. Importantly, deleting *EXO1* also reverses the growth defect of *mrc1Δ yku70Δ* mutants (Fig. 3B). These data suggest that Mrc1 protects uncapped telomeres from Exo1.

### 3.4 Effects of checkpoint mutations on *cdc13-1 mrc1Δ* and *yku70Δ mrc1Δ* growth

To understand if checkpoint pathways are activated in *mrc1Δ* strains after telomere uncapping, we wanted to combine *cdc13-1 mrc1Δ* and *yku70Δ mrc1Δ* strains with checkpoint mutations. A genetic screen has revealed that *mrc1Δ* is synthetically lethal with *rad9Δ, rad17Δ* or *rad24Δ* checkpoint mutations [24]. Consistent with these results we were unable to recover viable offspring carrying *mrc1Δ* in combinations of any of these checkpoint genes (data not shown). However, we were able to combine *mrc1Δ* with *rad53Δ* and *chk1Δ*, encoding two downstream checkpoint kinases (analogues of human Chk2 and Chk1, respectively).

**Fig. 4:**
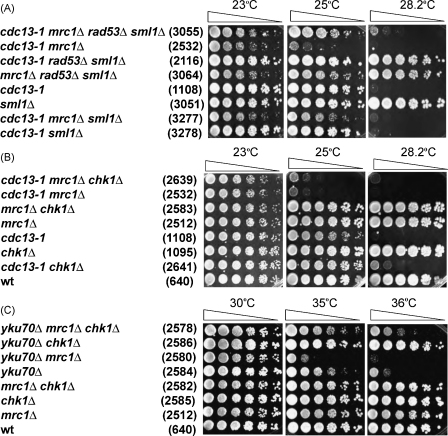
Differential suppression of *cdc13-1 mrc1Δ* and *yku70Δ mrc1Δ* growth defects by checkpoint mutations. Colonies were plated as described in Fig. 1. The relevant genotypes are indicated on the left, and strain numbers are shown in parentheses.

When *cdc13-1* mutants grow at non-permissive temperatures Rad53 and Chk1 dependent parallel pathways are activated [36,45,46]. We deleted *RAD53* or *CHK1* and examined the effects in *cdc13-1 mrc1Δ* mutants. Deletion of *RAD53* requires simultaneous deletion of *SML1* to obtain viable spores. Sml1 is a small protein that inhibits the activity of ribonucleotide reductase (RNR) which catalyzes the rate-limiting step of de novo dNTP synthesis [47]. Normally Sml1 is degraded in a Rad53-dependent manner during S phase [48]. We found that removal of *RAD53* and *SML1* improved the growth of *cdc13-1 mrc1*Δ strains (Fig. 4A). However, deletion of *SML1* alone (*cdc13-1 mrc1*Δ *sml1*Δ) also rescued the growth defects associated with *MRC1* deletion (Fig. 4A). Thus, we were unable to observe any strong role for Rad53, in maintaining vitality of *cdc13-1 mrc1Δ* mutants, other than in degrading Sml1. We suggest that the reason that deleting Sml1 improves the growth of *cdc13-1 mrc1Δ* strains is that increased ribonucleotide reductase activity may stabilise the replication forks.

Removal of Chk1, like removal of Rad53, does not rescue growth of *cdc13-1 mrc1Δ* strains, indicating that Chk1 does not inhibit growth of these mutants (Fig. 4B). Therefore, we find no evidence that inactivating DNA damage checkpoint pathways improves growth of *cdc13-1 mrc1Δ* mutants.

A *CHK1* deletion strongly rescues growth of *yku70Δ mrc1Δ* mutants at restrictive temperatures (Fig. 4C) similarly to its effect in *yku70Δ* mutants [36]. Thus, *mrc1Δ yku70Δ* uncapped telomeres qualitatively behave like *yku70Δ* uncapped mutants. The effects of *chk1Δ* in *yku70Δ mrc1Δ* and *cdc13-1 mrc1Δ* strains are consistent with earlier findings showing that the Chk1-dependent pathway is more important in *yku70Δ* mutants [36,45].

### 3.5 Mrc1 is not required for the cell cycle arrest after *cdc13-1* uncapping

**Fig. 5:**
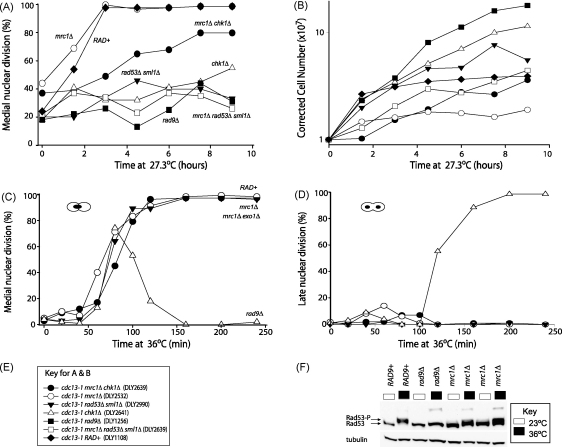
Mrc1 does not contribute to checkpoint activation after *cdc13-1* dependent telomere uncapping. (A and B) 7 *cdc13-1* strains, whose genotypes are shown in e, were switched from 23 °C to 27.3 °C and their cell cycle position and growth were monitored, as described in Section 2. (C and D) *cdc15-2 bar1* strains with additional mutations [DLY2646 (*cdc13-1 mrc1Δ*), DLY3071 (*cdc13-1 mrc1Δ exo1Δ*), DLY1468 (*cdc13-1*), DLY1470 (*cdc13-1 rad9Δ*)] were synchronised with α-factor, released from G1 to non-permissive temperature (36 °C) to induce telomere uncapping and cell cycle position was measured, after cells were fixed in 70% ethanol and stained with DAPI. (E) Genotypes of strains used for the asynchronous cultures demonstrated in (a and b) are shown. (F) Western blot demonstrating Rad53 phosphorylation in various *cdc13-1* strains with the additional mutations indicated [DLY1108 (*cdc13-1 RAD+*), DLY1256 (*cdc13-1 rad9Δ*), DLY2532 (*cdc13-1 mrc1Δ*), DLY2533 (*cdc13-1 mrc1Δ*)]. Cultures were grown overnight at 23 °C, diluted in the morning and divided in two. While still growing exponentially, the temperature was raised to 36 °C in one of the aliquots and samples were taken 2 h later and processed for Western blots. Anti-Tubulin was used as a loading control.

To directly test whether Mrc1 plays a checkpoint role in *cdc13-1* strains, *cdc13-1* strains with additional mutations were first grown at the semi-permissive temperature 27.3 °C. Strains defective in telomere capping (*cdc13-1*) arrest at medial nuclear division before entry to anaphase after 3 h at 27.3 °C (Fig. 5A). As expected, when the checkpoint is compromised in *cdc13-1 chk1Δ*, *cdc13-1 rad9Δ* or *cdc13-1 rad53Δ* cells, no accumulation at medial nuclear division is observed over 9 h (Fig. 5A). In contrast, *cdc13-1* and *cdc13-1 mrc1Δ* strains rapidly accumulate at medial nuclear division and within 3 h more than 90% of cells are arrested at this point. Consistent with these conclusions cell numbers stopped increasing in *cdc13-1* and *cdc13-1 mrc1Δ* strains, but continued to increase in the other strains (Fig. 5B). We conclude that Mrc1 is not required for the checkpoint response to *cdc13-1* dependent telomere uncapping.

Rad53 and Chk1 are components of parallel checkpoint pathways that respond to *cdc13-1* induced telomere uncapping [45,46]. It appears that the Rad53 pathway is more important for arrest of *cdc13-1 mrc1Δ* mutants because 80% of *cdc13-1 mrc1Δ chk1Δ* cells have arrested at medial nuclear division by 7.5 h, whereas there is no arrest of *cdc13-1 mrc1Δ rad53Δ* cells.

To test the role of Mrc1 in checkpoint control in a single cell cycle we combined *mrc1Δ* with *cdc13-1 cdc15-2* and *bar1* mutations. Over many years we and others have used these mutations to determine the effects of checkpoint proteins in responding to telomere uncapping [13,39,40,45]. Bar1 encodes a protease that degrades the mating pheromone α-factor. Cells bearing the *bar1* mutation can efficiently arrest in G1 phase of the cell cycle with low levels of α-factor. Cdc15 is necessary for mitotic exit. At 36 °C, *cdc13-1 cdc15-2 bar1* control strains, released from alpha factor arrest, accumulate at medial nuclear division due to *cdc13-1-*dependent telomere uncapping. However, if cells have escaped the G2/M checkpoint, like *cdc13-1 rad9Δ cdc15-2 bar1* strains, they arrest at late nuclear division due to *cdc15-2* and they are unable to proceed to the next cycle. The *cdc15-2* dependent cell cycle arrest helps in two ways, it ensures that DNA damage checkpoint defects are easily quantified because cells with checkpoint defects accumulate at a later stage of the cell cycle and that DNA damage caused by *cdc13-1* is not amplified during new rounds of DNA replication.

*cdc13-1 cdc15-2 bar1* strains with additional mutations were arrested with α-factor, then released from G1 and transferred to a non-permissive temperature to induce telomere uncapping and the cell cycle position was monitored. Fig. 5C shows that in contrast to *cdc13-1 rad9Δ* strains *cdc13-1* and *cdc13-1 mrc1Δ* strains arrest at medial nuclear division with similar kinetics at 36 °C, supporting the idea that Mrc1 does not play a role in checkpoint activation after *cdc13-1* telomere uncapping. Interestingly, *cdc13-1 mrc1Δ exo1Δ* remain arrested at medial nuclear division (Fig. 5C and D) which contrasts to the behaviour of *cdc13-1 exo1Δ* that begin to escape arrest during the 4-h period in analogous experiments [39]. This difference most likely reflects the fact that *cdc13-1 mrc1Δ exo1Δ* mutants have more severe telomere capping defect than *cdc13-1 exo1Δ* mutants and therefore activate a stronger checkpoint signal.

In response to both replication stress and DNA damage, activation of the checkpoint machinery induces phosphorylation and activation of Rad53 kinase. Therefore, we addressed the role of Mrc1 in Rad53 phosphorylation after telomere uncapping. *cdc13-1* strains were exposed to 36 °C for 2 h and Rad53 phosphorylation was measured by Western blot. Rad53 phosphorylation is observed in *cdc13-1 mrc1Δ* strains but not in *cdc13-1 rad9Δ* mutants (Fig. 5F), confirming a previous study [23]. We conclude that activation of Rad53 after *cdc13-1* dependent uncapping at non-permissive temperatures is Rad9-dependent but Mrc1-independent.

### 3.6 Mrc1 contributes to telomere length regulation

**Fig. 6:**
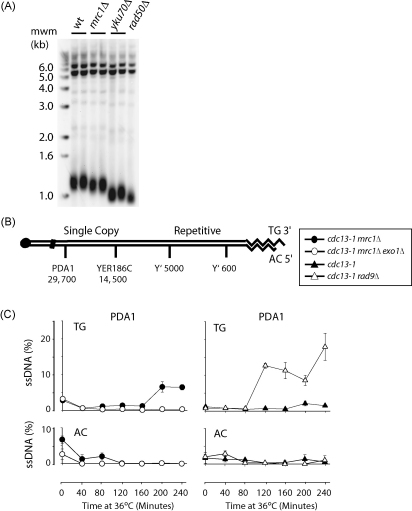
Mrc1 protects telomeres from shortening and inhibits ssDNA generation at uncapped telomeres. (A) Southern blot where the telomere length of various strains was examined. DNA was extracted from strains grown at 23 °C in liquid YEPD. Strains used were DLY640 (wild type), DLY641 (wild type), DLY2512 (*mrc1*Δ), DLY2709 (*mrc1*Δ), DLY1366 (*yku70*Δ), DLY2584 (*yku70*Δ) and DLY1091 (*rad50*Δ). (B) Schematic figure to show the *PDA1* locus on chromosome V. (C) ssDNA measurements in the single copy locus of *cdc15-2 bar1* strains with the additional mutations indicated; DLY2646 (*cdc13-1 mrc1Δ*), DLY3071 (*cdc13-1 mrc1Δ exo1Δ*), DLY1468 (*cdc13-1*), DLY1470 (*cdc13-1 rad9Δ*). Samples were taken, during synchronous cultures, at the indicated time points after cultures were released from G1 arrest and transferred to non-permissive temperatures (36 °C). DNA preparations were assessed by quantitative amplification of ssDNA (QAOS) [37] to measure ssDNA on the TG and AC strand at *PDA1* locus. The error bars represent the standard error of the mean calculated from three independent measurements of each sample.

If Mrc1 plays a protective role at telomeres, this predicts that strains lacking Mrc1 may have short telomeres. Fig. 6A shows that absence of Mrc1 results in shorter telomeres, compared to the wild type. However, the telomere length defects of *mrc1Δ* mutants are not as severe as in *rad50Δ* or *yku70Δ* mutants, and this may help explain why *cdc13-1 mrc1Δ* cells grow better than *cdc13-1 rad50Δ* cells (Supplementary Fig. 1). Our experiment is consistent with replication proteins having an important role in telomere length regulation [49–52].

### 3.7 Mrc1 protects telomeres from extended ssDNA accumulation

All our data suggest an important role of Mrc1 in telomere capping but no role in cell cycle arrest. To directly test the role of Mrc1 in telomere capping, we measured ssDNA accumulation on the 3′ TG strand, at *PDA1*, a single copy locus approximately 30 kb away from the right end of chromosome V in *cdc13-1* strains (Fig. 6B). *cdc13-1* strains were synchronised, as in Fig. 5C and D in order to follow the effects of Mrc1 on ssDNA accumulation at non-permissive temperatures within a single cell cycle. We find that *cdc13-1 mrc1Δ* mutants, like *cdc13-1 rad9Δ* strains, accumulate more 3′ TG ssDNA at *PDA1*, 30 kb from the uncapped telomere, compared to *cdc13-1* strains (Fig. 6C) [39]. This shows that Mrc1, like Rad9, protects *cdc13-1* mutants from ssDNA production. Consistent with our conclusion, increased ssDNA accumulation, closer to the telomere, in telomere repeats, was recently reported in *cdc13-1 mrc1Δ* and *yku70Δ mrc1Δ* mutants using both in gel and dot blot analyses [53]. Importantly, ssDNA production is reduced in *cdc13-1 mrc1Δ exo1Δ* strains in comparison to *cdc13-1 mrc1Δ* strains showing that Mrc1 protects uncapped telomeres from Exo1-dependent nuclease action. This ssDNA data is consistent with our finding that Exo1 contributes to the poor growth of *cdc13-1 mrc1Δ* and *yku70Δ mrc1Δ* mutants (Fig. 3). Taken together, we conclude that Mrc1 inhibits accumulation of Exo1-dependent ssDNA accumulation after telomere uncapping and, by this criterion, contributes to telomere capping.

## 4 Discussion

Our experiments demonstrate that Mrc1 contributes to the vitality of budding yeast cells with uncapped telomeres. Therefore, Mrc1 behaves differently from many other known checkpoint proteins such as Chk1, Mec1, Rad9, Rad17, Rad24 or Rad53, deletion of which improves the growth of *cdc13-1* mutants at semi-permissive temperatures [39,40]. The effect of Mrc1 is more similar to that of the MRX complex, another checkpoint complex with roles in telomere capping [36,42,54,55]. It seems that it is the role of Mrc1 at the replication fork that contributes to the vitality of telomere capping mutants, rather than its role in checkpoint activation. Our findings are in accordance with recent work that demonstrated a protective role of Mrc1 in cells with *cdc13-1* or *yku70Δ* uncapped telomeres or in telomerase deficient cells [53]. Additionally, our work demonstrates that the growth defects of *cdc13-1 mrc1Δ* and *yku70Δ mrc1Δ* mutants and enhanced ssDNA levels of *cdc13-1 mrc1Δ* strains are suppressed when the nuclease encoded by *EXO1* is deleted. Therefore, Mrc1 protects uncapped telomeres from Exo1.

Mrc1 is recruited to the replication machinery as DNA replication initiates and is required for normal rates of replication fork progression [17,18,22]. The biochemical role of Mrc1 in replication fork progression is unclear which makes it difficult to know its precise role in telomere capping. Mrc1 is also part of a replication-pausing complex formed when replication is arrested by the S phase poison hydroxyurea (HU), and required for replication fork restart after HU. However, this restart role for Mrc1 is not universal, since Mrc1 plays no role in replication restart after cells are treated with the alkylating agent MMS [22].

It is interesting that there is evidence from budding yeast, fission yeast and human cells that telomeric sequences contain DNA regions that slow or stall replication forks [15,56,57]. From this it seems plausible that telomeric DNA may be more dependent on proteins like Mrc1, which contribute to fork stability and restart, than other chromosomal regions. That is because the replication fork struggles to reach the end of the chromosome in *mrc1Δ* mutants where a telomere capping defect is observed.

Numerous studies on budding yeast mutants with DNA replication defects have demonstrated interactions between DNA replication and telomere structure. For example both *cdc17/pol1* and *cdc44/rfc1* (large subunit of replication factor C) mutants affect telomere length [49]. Here we show that budding yeast *mrc1*Δ mutants have short telomeres. In *S. cerevisiae*, *cdc17/pol1* mutants, encoding temperature sensitive DNA polymerase α, exhibit very long telomeres, high levels of telomeric ssDNA and elevated recombination at telomeres [50,51]. Interestingly, the B subunit of DNA polymerase α physically interacts with Stn1, which in turn interacts with Cdc13 [10,52]. This shows there is a very direct interaction between budding yeast telomere capping proteins and the replication fork machinery, and suggests that telomere capping is intimately linked with DNA replication. In this regard it is, perhaps, relevant that the 5′–3′ exonuclease Exo1 is involved in generating single stranded DNA at uncapped telomeres [36,39] and in processing stalled replication forks [58] and highlights the similarities between uncapped telomeres and stalled replication forks.

*cdc13-1* cells maintain a functional telomere cap (low levels of telomeric ssDNA), when released from G1 arrest into the S phase poison hydroxyurea. HU stalls replication forks and stops late origins of replication from firing. However, if the same *cdc13-1* cells are permitted to complete DNA replication, by removing the S phase poison HU, telomere uncapping occurs and high levels of ssDNA are observed [59]. Therefore, Cdc13-dependent telomere capping may depend on a coordinated interaction between the chromosome end, Cdc13/Stn1/Ten1 and the DNA replication fork. Further studies examining the interactions between telomeric DNA, the telomere cap and the replication fork will be necessary to better understand these interactions.
