# Supplementary material for: Mrc1 protects uncapped budding yeast telomeres from exonuclease EXO1
Source: DNA Repair (Amst). 2007 Nov 1;6(11):1607–17. doi: 10.1016/j.dnarep.2007.05.010 (PMC2077361; doi:10.1016/j.dnarep.2007.05.010)
Supplement: Supplementary file 1 [file mmc1.pdf]

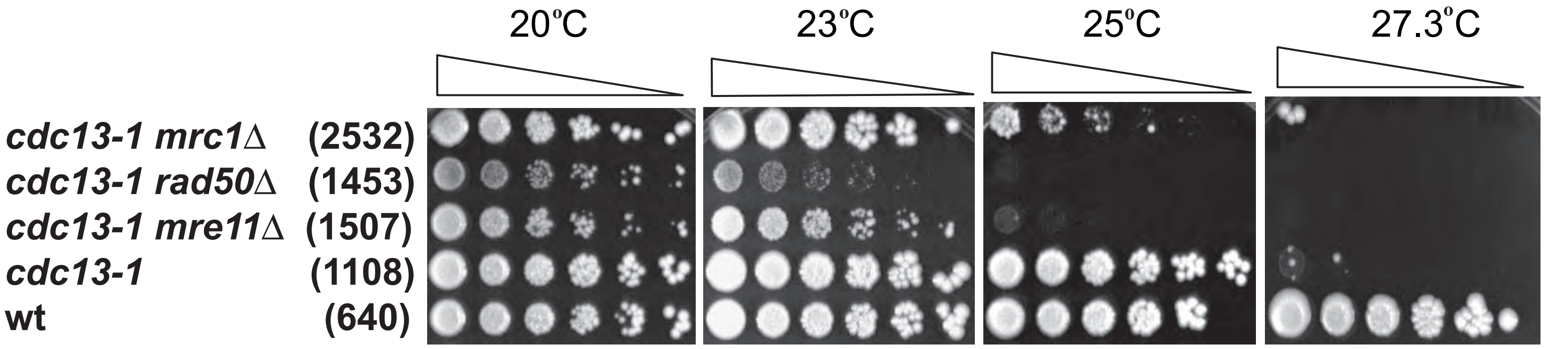

Tsolou, Supplementary Figure 1

Table 1: Yeast strains. * indicates strains generated in this study

Strain	Genotype	Gen Background	Source
640	*MATa ade2-1 trp1-1 can1-100 leu2-3,112 his3-11,15 ura3 GAL+ psi+ ssd1-d2 RAD5*	W303	R.Rothstein
641	*MATalpha ade2-1 trp1-1 can1-100 leu2-3,112 his3-11,15 ura3 GAL+ psi+ ssd1-d2 RAD5*	W303	R.Rothstein
1091	*MATa rad50::hisG::URA3 ade2-1 trp1-1 can1-100 leu2-3,112 his3-11,15 ura3 GAL+ psi+ ssd1-d2 RAD5*	W303	640
1095	*MATa chk1::HIS3 ade2-1 trp1-1 can1-100 leu2-3,112 his3-11,15 ura3 GAL+ psi+ ssd1-d2 RAD5*	W303	640
1108	*MATa ade2-1 trp1-1 can1-100 leu2-3,112 his3-11,15 ura3 GAL+ psi+ ssd1-d2 RAD5 cdc13-1-int*	W303	640
1256	*MATalpha cdc13-1int rad9::HIS3 RAD5 ade2-1 trp1-1 can1-100 leu2-3,112 his3-11,15 ura3 GAL+ psi+ ssd1-d2*	W303	662x1218
1296	*MATa exo1::LEU2 cdc13-1-int ade2-1 trp1-1 can1-100 leu2-3,112 his3-11,15 ura3 GAL+ psi+ ssd1-d2 RAD5*	W303	1272x1230
1366	*MATalpha yku70::HIS3 ade2-1 trp1-1 can1-100 leu2-3,112 his3-11,15 ura3 GAL+ psi+ ssd1-d2 RAD5*	W303	1308x1284
1408	*MATa ade2-1 trp1-1 can1-100 leu2-3,112 his3-11,15 ura3 GAL+ psi+ ssd1-d2 RAD5 yku70::HIS3 exo1::LEU2*	W303	1273x1364
1453	*MATalpha rad50::hisG::URA3 cdc13-1 int ade2-1 trp1-1 can1-100 leu2-3,112 his3-11,15 ura3 GAL+ psi+ ssd1-d2 RAD5*	W303	1091*1260
1468	*MATa bar1::hisG RAD+ ade2-1 trp1-1 can1-100 leu2-3,112 his3-11,15 ura3 GAL+ psi+ ssd1-d2 RAD5 cdc15-2 int cdc13-1 int*	W303	1436x1438
1470	*MATa bar1::hisG rad9::HIS3 ade2-1 trp1-1 can1-100 leu2-3,112 his3-11,15 ura3 GAL+ psi+ ssd1-d2 RAD5 cdc15-2 int cdc13-1 int*	W303	1436x1438
1507	*MATalpha cdc13-1int mre11::hisG::URA3 RAD5 ade2-1 trp1-1 can1-100 leu2-3,112 his3-11,15 ura3 GAL+ psi+ ssd1-d2*	W303	1331*1259
1584	*MAT alpha mfa::MFA1pr-HIS3 can1 ura3 leu2 his3 lys2*	S288C	Dan Durocher
2116	*MATalpha rad53::HIS3 sml1::Kan exo1::LEU2 cdc13-1int cdc15-2 int bar1 RAD5 ade2-1 trp1-1 can1-100 ura3 GAL+ psi+ ssd1-d2*	W303	1765x1431
2512*	*MATa ade2-1 trp1-1 can1-100 leu2-3,112 his3-11,15 ura3 GAL+ psi+ ssd1-d2 RAD5 mrc1::KANMX6*	W303	640
2532*	*MATalpha mrc1::KANMX6 cdc13-1 int ade2-1 trp1-1 can1-100 leu2-3,112 his3-11,15 ura3 GAL+ psi+ ssd1-d2 RAD5*	W303	2512 x 1297
2533*	*MATa mrc1::KANMX6 cdc13-1 int ade2-1 trp1-1 can1-100 leu2-3,112 his3-11,15 ura3 GAL+ psi+ ssd1-d2 RAD5*	W303	2512 x 1297
2534*	*MATa mrc1::KANMX6 cdc13-1 int exo1::LEU2 ade2-1 trp1-1 can1-100 leu2-3,112 his3-11,15 ura3 GAL+ psi+ ssd1-d2 RAD5*	W303	2512 x 1297
2535*	*MATalpha mrc1::KANMX6 cdc13-1 int exo1::LEU2 ade2-1 trp1-1 can1-100 leu2-3,112 his3-11,15 ura3 GAL+ psi+ ssd1-d2 RAD5*	W303	2512 x 1297
2578*	*MATalpha mrc1::KANMX6 yku70::LEU2 chk1::HIS3 ade2-1 trp1-1 can1-100 leu2-3,112 his3-11,15 ura3 GAL+ psi+ ssd1-d2 RAD5*	W303	2512x1266
2580*	*MATa mrc1::KANMX6 yku70::LEU2 ade2-1 trp1-1 can1-100 leu2-3,112 his3-11,15 ura3 GAL+ psi+ ssd1-d2 RAD5*	W303	2512x1266

2582*	*MATalpha mrc1::KANMX6 chk1::HIS3 ade2-1 trp1-1 can1-100 leu2-3,112 his3-11,15 ura3 GAL+ psi+ ssd1-d2 RAD5*	W303	2512x1266
2583*	*MATa mrc1::KANMX6 chk1::HIS3 ade2-1 trp1-1 can1-100 leu2-3,112 his3-11,15 ura3 GAL+ psi+ ssd1-d2 RAD5*	W303	2512x1266
2584*	*MATalpha yku70::LEU2 ade2-1 trp1-1 can1-100 leu2-3,112 his3-11,15 ura3 GAL+ psi+ ssd1-d2 RAD5*	W303	2512x1266
2585*	*MATalpha chk1::HIS3 ade2-1 trp1-1 can1-100 leu2-3,112 his3-11,15 ura3 GAL+ psi+ ssd1-d2 RAD5*	W303	2512x1266
2586*	*MATalpha yku70::LEU2 chk1::HIS3 ade2-1 trp1-1 can1-100 leu2-3,112 his3-11,15 ura3 GAL+ psi+ ssd1-d2 RAD5*	W303	2512x1266
2639*	*MATa mrc1::KANMX6 chk1::HIS3 cdc13-1 int ade2-1 trp1-1 can1-100 leu2-3,112 his3-11,15 ura3 GAL+ psi+ ssd1-d2 RAD5*	W303	2533x2585
2641*	*MATalpha chk1::HIS3 cdc13-1 int ade2-1 trp1-1 can1-100 leu2-3,112 his3-11,15 ura3 GAL+ psi+ ssd1-d2 RAD5*	W303	2533x2585
2646*	*MATa mrc1::KanMX6 EXO1 bar1::hisG ade2-1 trp1-1 can1-100 leu2-3,112 his3-11,15 ura3 GAL+ psi+ ssd1-d2 RAD5 cdc15-2 int cdc13-1 int*	W303	2619x1431
2709*	*MATalpha ade2-1 trp1-1 can1-100 leu2-3,112 his3-11,15 ura3 GAL+ psi+ ssd1-d2 RAD5 mrc1::KANMX6*	W303	2512x1297
2990*	*MATalpha rad53::HIS3 sml1::KanMX6 cdc13-1 RAD5 ade2-1 trp1-1 can1-100 ura3 GAL+ psi+ ssd1-d2*	W303	2930x2116
3051*	*MATalpha sml1::KanMX6 RAD5 ade2-1 trp1-1 can1-100 ura3 GAL+ psi+ ssd1-d2*	W303	2930x2116
3055*	*MATalpha mrc1::URA3 rad53::HIS3 sml1::KanMX6 cdc13-1 RAD5 ade2-1 trp1-1 can1-100 ura3 GAL+ psi+ ssd1-d2*	W303	2993x1296
3062*	*MATalpha mrc1::KANMX6 yku70::HIS3 exo1::LEU2 ade2-1 trp1-1 can1-100 leu2-3,112 his3-11,15 ura3 GAL+ psi+ ssd1-d2 RAD5*	W303	2709x1408
3064*	*MATa mrc1::URA3 rad53::HIS3 sml1::KanMX6 RAD5 ade2-1 trp1-1 can1-100 ura3 GAL+ psi+ ssd1-d2*	W303	3052x2991
3071*	*MATa mrc1::KanMX6 exo1::LEU2 bar1::hisG ade2-1 trp1-1 can1-100 leu2-3,112 his3-11,15 ura3 GAL+ psi+ ssd1-d2 RAD5 cdc15-2 int cdc13-1 int*	W303	2619x1431
3277*	*MATalpha mrc1::URA3 sml1::KanMX6 cdc13-1 CDC15+ RAD5 ade2-1 trp1-1 can1-100 ura3 GAL+ psi+ ssd1-d2*	W303	2993x1296
3278*	*MATa sml1::KanMX6 cdc13-1 CDC15+ RAD5 ade2-1 trp1-1 can1-100 ura3 GAL+ psi+ ssd1-d2*	W303	2993x1296
3393	*MATa LEU2::cdc13-1 int::URA3 mfa::MFA1pr-HIS3 ura30 leu20 his31 lys20 tof1::KANMX*	S288C (C4B)	Dan Durocher
3394	*MATa LEU2::cdc13-1 int::URA3 mfa::MFA1pr-HIS3 ura30 leu20 his31 lys20 tof1::KANMX*	S288C (FIA)	Dan Durocher
3395	*MATa LEU2::cdc13-1 int::URA3 mfa::MFA1pr-HIS3 ura30 leu20 his31 lys20 mrc1::KANMX*	S288C (C4B)	Dan Durocher
3396	*MATa LEU2::cdc13-1 int::URA3 mfa::MFA1pr-HIS3 ura30 leu20 his31 lys20 mrc1::KANMX*	S288C (FIA)	Dan Durocher
3397	*MATa LEU2::cdc13-1 int::URA3 mfa::MFA1pr-HIS3 ura30 leu20 his31 lys20 csm3::KANMX*	S288C (C4B)	Dan Durocher
3398	*MATa LEU2::cdc13-1 int::URA3 mfa::MFA1pr-HIS3 ura30 leu20 his31 lys20 csm3::KANMX*	S288C (FIA)	Dan Durocher
3402	*MATa LEU2::cdc13-1 int::URA3 mfa::MFA1pr-HIS3 ura30 leu20 his31 lys20 his3::KANMX*	S288C (C4B)	Dan Durocher
